# Characterization and Classification of Direct and Commercial Strawberry Beverages Using Absorbance–Transmission and Fluorescence Excitation–Emission Matrix Technique

**DOI:** 10.3390/foods11142143

**Published:** 2022-07-20

**Authors:** Ewa Sikorska, Przemysław Nowak, Katarzyna Pawlak-Lemańska, Marek Sikorski

**Affiliations:** 1 Department of Technology and Instrumental Analysis, Institute of Quality Science, Poznan University of Economics and Business, al. Niepodległosci 10, 61-875 Poznan, Poland; katarzyna.pawlak-lemanska@ue.poznan.pl; 2 Faculty of Chemistry, Department of Spectroscopy and Magnetism, Adam Mickiewicz University in Poznan, ul. Uniwersytetu Poznanskiego 8, 61-614 Poznan, Poland; nowapr@gmail.com (P.N.); sikorski@amu.edu.pl (M.S.)

**Keywords:** food quality, spectroscopy, chemometrics, fruit juice, multivariate analysis

## Abstract

The subject of this study was to characterize the absorption and fluorescence spectra of various types of strawberry beverages and to test the possibility of distinguishing between direct juices and pasteurized commercial products on the basis of their spectral properties. An absorbance and transmission excitation–emission matrix (A-TEEM^TM^) technique was used for the acquisition of spectra. The obtained spectra were analyzed using chemometric methods. The principal component analysis (PCA) revealed differences in both the absorption spectra and excitation–emission matrices (EEMs) of two groups of juices. The parallel factor analysis (PARAFAC) enabled the extraction and characterization of excitation and emission profiles and the relative contribution of four fluorescent components of juices, which were related to various groups of polyphenols and nonenzymatic browning products. Partial least squares–discriminant analysis (PLS-DA) models enabled 100% correct class assignment using the absorption spectra in the visible region, unfolded EEMs, and set of emission spectra with excitation at wavelengths of 275, 305, and 365 nm. The analysis of variable importance in projection (VIP) suggested that the polyphenols and nonenzymatic browning products may contribute significantly to the differentiation of commercial and direct juices. The results of the research may contribute to the development of fast methods to test the quality and authenticity of direct and processed strawberry juices.

## 1. Introduction

The strawberry (*Fragaria* × *ananassa*) is a popular fruit with attractive sensory attributes and a high content of nutrient and bioactive non-nutrient components. In particular, it is an important source of dietary fiber, vitamin C, folate, polyphenols, and minerals [1]. Consumption of it is associated with several health benefits, such as the prevention of oxidative stress, inflammations, some types of cancers, type-2 diabetes, obesity, and cardiovascular and neurodegenerative diseases. The health-promoting effects of strawberry intake are mainly related to polyphenols, which exhibit antioxidant, anti-inflammatory, anti-hypertensive, anti-hyperglycemic, and cell-protecting effects [2]. In addition, polyphenols are one of the determinants of the attractive sensory attributes of strawberries, including color, flavor, astringency, and hardness [3].

Strawberries are economically and commercially important due to their high consumption. They are consumed fresh and processed into various products because of their very short shelf-life and seasonality. Important processed products are various beverages, including juices, nectars, and syrups.

In order to ensure the high quality of fruit beverages, there is a need to develop fast and direct methods that allow the analysis of a large number of samples throughout the production and distribution chain, in an economically efficient and environmentally friendly manner.

One of the important aspects of the quality of the juice is its authenticity. Juices are often the subject of fraudulent economically motivated adulteration (EMA) practices. These practices include: dilution with water; substitution with cheap ingredients; the addition of sugar, organic acid, artificial sweetener, flavor, or colorant; and the addition of by-products or less expensive juice. For example, juice from concentrate (FC) is added to not-from-concentrate (NFC) juice, or these two types of juices are mislabeled.

The techniques used for quality and authenticity testing include targeted and untargeted analysis. Targeted analysis is based on the identification and quantification of specific compounds, which serve as markers of the quality or authenticity of the product. Untargeted analysis focuses on the acquisition of non-selective “chemical fingerprints” of the samples and their analysis with multivariate methods [4]. Spectroscopic techniques are frequently used as a fingerprinting tool in the quality and authenticity control of foods, including berry fruits and juices [5,6,7,8].

The spectroscopic method most commonly used in food quality control is near–infrared (NIR) spectroscopy. However, in the case of fruit juices, the NIR spectra are dominated by intense absorption bands from water, which hide the lower absorption of the minor components of the juice. Thus, other spectroscopic techniques are tested to study this group of products. A growing number of studies demonstrate that molecular absorption and fluorescence spectroscopy coupled with multivariate analysis has been successfully used as a fingerprinting technique in the quality evaluation of various food products, including fruit juice [7].

Absorption spectroscopy in the ultraviolet and visible (UV–vis) region enables the observation of chromophores with high electron density, including pigments present in juice [9]. Many of the food components are fluorescent, and they contribute to the specific autofluorescence of products that may be used to evaluate various aspects of quality. Fluorescence was successfully used to evaluate various properties of fruit juices and extracts, including: apple [10,11], peach [12] plum [13,14], avocado [15], orange [16], and berry fruit [17]. In most of these studies, the fluorescence of the juices or extracts studied was associated with the presence of polyphenolic compounds.

Due to the complex nature of food autofluorescence, a multidimensional technique such as excitation–emission matrix spectroscopy is often used to study foodstuffs. The excitation–emission matrices (EEMs), known also as total fluorescence spectra, represent the emission intensity as a function of both excitation and emission wavelengths and provide overall comprehensive characteristics of studied samples determined by both absorption and fluorescence properties. An analysis of EEMs arrays for the set of samples using the multiway parallel factor analysis (PARAFAC) method enables the extraction of excitation and emission profiles and relative concentrations of fluorescent components. The EEMs may also be used to build regression or classification models.

Recently, a new absorbance–transmission and fluorescence excitation–emission matrix technique (A-TEEM^TM^) was developed, which enables the simultaneous acquisition of UV–vis absorption spectra and EEMs. One of the advantages of this technique is the rapid acquisition of EEMs. Another important advantage is that the absorption spectra provide complementary information to the EEMs, as they contain signals not only from fluorophores but also from all other substances absorbing in the UV–vis region in the sample under study [18]. Furthermore, the measured absorbance enables the correction of inner filter effects (IFE), which occur in samples with a high optical density [19] and result in a deviation from the linear relationship between the fluorescence intensity and concentration, leading to the distortion of the shape of the emission bands and changes to their relative intensity in the EEMs. The A-TEEM^TM^ technique is successfully used for water analysis and has been applied to the study of wine [20,21,22] and beer [23].

The use of fingerprint techniques for quality control requires knowledge of the spectral profiles of the products. The objectives of this study were the characterization of spectral properties and the discrimination between direct fresh juice and commercial pasteurized beverages obtained from strawberries, using an A-TEEM^TM^ technique coupled with chemometrics.

## 2. Materials and Methods

### 2.1. Materials

The subjects of this study were freshly squeezed juices (*n* = 15, D1–D15) and commercial processed beverages obtained from strawberry fruits (*Fragaria × ananassa* Duch.) (*n* = 20, P1–P20). The detailed characteristics of samples studied are presented in Table S1 (Supplementary Materials).

Direct fresh juices were obtained from fruits of several varieties: Azja (samples D1–D5), Florence (samples D6–D8), Florentina (sample D9), Honeoye (sample D10), Korona (sample D11), Romina (sample D12), Rumba (D13–D15). The fruits were washed and dried, and the juice was squeezed using the low-speed squeezer Hurom HR (Hurom Co., Ltd., Gimhae, South Korea).

Twenty processed commercial beverages covered various categories of products available on the market, including those obtained only from strawberries and with the addition of other fruits, including: juices, nectars, drinks (P1–P10), and syrups (P11–P20). They were produced differently, depending on the juice category, and also came from different producers.

The juices were stored at −20 °C and, before analysis, were thawed and equilibrated at room temperature.

### 2.2. Acquisition of Absorption and EEMs

The absorption and fluorescence spectra were measured using the Aqualog^®^ spectrofluorimeter (Horiba Instruments Inc., Montpellier, France). The instrument includes a Si photodiode absorbance detector and an emission detector comprised of a thermoelectrically cooled back-illuminated CCD and spectrograph [18].

The absorption and fully corrected EEMs were simultaneously collected by scanning the excitation wavelengths in the range of 240–800 nm with a resolution of 5 nm and recording the emission in the wavelength range of 242–824 nm with an increment of 4.66 nm. The slit widths of both the excitation and emission monochromators were 5 nm, and the integration time was 0.2 s. Measurements were made in quartz cuvettes with an optical path length of 1 cm. The juices for the measurements were centrifuged (14,100× *g* for 5 min) (Eppendorf AG, Hamburg, Germany) and diluted with distilled water (1:100). Three measurements were made for each sample. The EEMs were corrected for the influence of inner filter effects (IFE), and the Rayleigh scattering signal was removed prior to the analysis.

### 2.3. Data Analysis

The absorption spectra were arranged for analysis in a matrix. The EEMs were arranged into three-way arrays or unfolded into matrix along the sample mode (uEEMs). Additionally, individual emission spectra measured at the particular excitation wavelength were used for analysis.

Parallel factor analysis (PARAFAC) was used to decompose EEMs into the contribution of individual fluorescent components [24]. PARAFAC analysis was performed for the EEMs presented in a three-dimensional array with dimensions of 35 × 64 × 29 elements (number of samples × number of emission wavelengths × number of excitation wavelengths). The optimal number of components in the PARAFAC models was selected based on the explained variance and the core consistency diagnostic. The mean contents of four groups of fluorophores were analyzed using the Student’s *t*-test.

Principal component analysis (PCA) was performed on UV–vis absorption spectra and uEEMs. Absorption spectra in the range of 240–800 nm were used for analysis and presented in the form of a matrix with dimensions of 35 × 113 (number of samples × number of absorption wavelength). The excitation–emission matrices in the range of excitation wavelengths of 260–400 nm and emission wavelengths of 310–600 nm were selected for analysis and were presented in the form of a 35 × 1856 matrix, defined by the number of samples × (number of emission wavelengths multiplied by the number of excitation wavelengths). The cross-validation method was used to check the models.

The partial least squares–discriminant analysis (PLS-DA) was used to develop classification models for two classes of products—direct and commercial processed beverages [25]. PLS-DA analysis was performed for entire UV–vis absorption spectra and their different ranges (UV and vis), entire uEEMs, scores obtained from PARAFAC analysis, and sets of selected emission spectra. The **X** matrix represented spectral data. The **Y** matrix consisted of two columns containing information on the assignment of a sample to a specific class. Cross-validation was used to verify the PLS-DA models. The performance of the classification models was determined on the basis of sensitivity, specificity, and misclassification error. Sensitivity was defined as the proportion of positive cases that were correctly identified. Specificity was defined as the proportion of negative cases that were classified correctly. The misclassification error indicated the proportion of samples which were incorrectly classified. These parameters were calculated using Equations (1)–(3), respectively:

(1) $$\text{Sensitivity}=\frac{TP}{TP+FN}$$

(2) $$\text{Specificity}=\frac{TN}{TN+FP}$$

(3) $$\text{Misclassification error}=\frac{FP+FN}{TP+TN+FP+FN}$$

where *TP*—true positive, *TN*—true negative, *FP*—false positive, *FN*—false negative cases.

Variable importance in projection (VIP) was used to estimate the importance of variables used in a PLS-DA model [26]. VIP provides a measure of the explanation of the variance of **X** by each of the variables and, simultaneously, of the correlation of **X** with **Y**. A variable with a VIP score close to or greater than unity was considered important in a model.

Multivariate data analysis was performed using Solo v. 5.0.1 software (Eigenvector Research Inc., Wenatchee, WA, USA); the *t*-test was performed using OriginPro 2020 (OriginLab Corporation, Northampton, MA, USA).

## 3. Results and Discussion

### 3.1. Absorption Spectra and Excitation–Emission Matrices of Studied Juices

The subjects of the study were the characterization of the UV–vis absorption and fluorescence properties of various sets of strawberry beverages. We investigated juices prepared in the laboratory directly squeezed from strawberry fruits and commercial beverages obtained from strawberries. The characteristics of the samples studied are presented in Table S1 (Supplementary Materials). To ensure product variability, the direct juices were obtained from fruits of seven varieties and collected in different seasons and locations. The commercial beverages were selected from various categories of products available on the market, including juices and drinks, nectars, and syrups. We tested products made only from strawberries, as well as beverages with the addition of other fruits and ingredients. 

**Figure 1:**
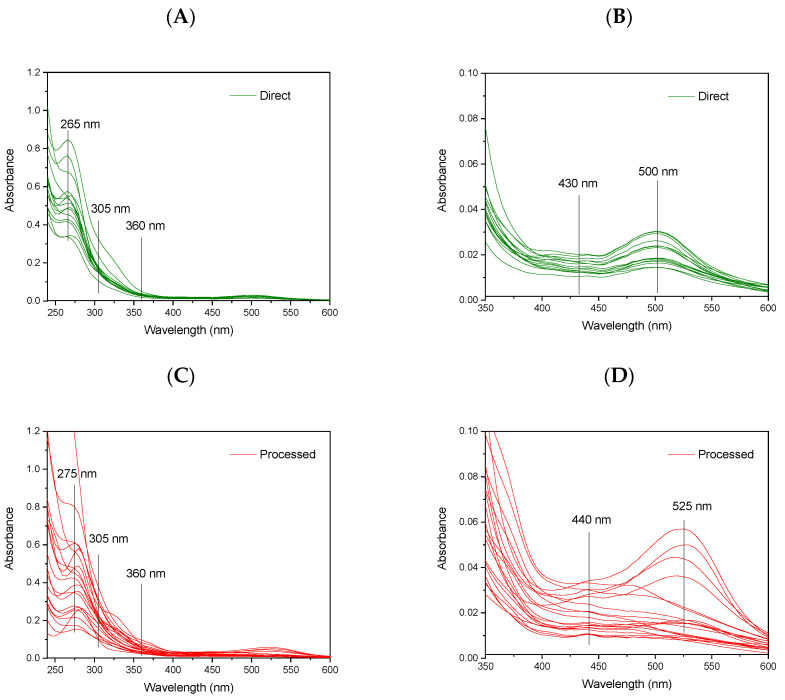
The absorption spectra of direct and processed strawberry beverages: Spectra of direct juices in the entire UV–vis region (**A**) and enlarged spectra in vis region (**B**). Spectra of processed beverages in the entire UV–vis region (**C**) and enlarged spectra in vis region (**D**).

The absorption spectra and EEMs were simultaneously recorded for each diluted juice using an A-TEEM^TM^ technique. Figure 1A shows the absorption spectra of the direct juices in the UV–vis region. Figure 1B presents the enlarged vis region of these spectra. Figure 1C,D present the respective spectra for the processed commercial beverages.

The absorption spectra of all the studied juices exhibit intense bands in the UV range and relatively less intense absorption bands in the vis range. The spectra of the fresh direct juices show a high similarity in the position and shape of the absorption bands and differ mainly in the intensity of the absorption. On the contrary, the spectra of the commercial juices are characterized by a greater diversity in both the position and shape of the absorption bands as well as in the intensity of the absorption. Due to the low selectivity of the UV–vis absorption technique, overlapped signals from the compounds of various groups contribute to the observed spectra. Based on the literature data, only tentative assignments of the origin of absorption bands in the UV and vis region can be made. In the UV range, the direct juices exhibited an intense absorption band with a maximum at about 265 nm. The absorption bands of the processed beverages show a maximum at 275 nm. Several compounds, including polyphenols and vitamins, may contribute to the observed absorption of the direct and processed beverages in this region. The main classes of phenolic compounds present in strawberries include flavonoids, represented mainly by anthocyanins, with a minor contribution of flavonols and flavanols, followed by hydrolyzable tannins, including ellagitannins and gallotannins, hydroxybenzoic acids, and hydroxycinnamic acids, and proanthocyanidins as minor constituents [1]. For example, in the ultra-performance liquid chromatography–photodiode array–fluorescence (UPLC–PDA–FL) analysis of cloudy strawberry juices, the wavelengths in the UV region were used for the detection of several groups of phenolic compounds. In particular, flavan-3-ols and ellagitannins were detected based on absorption at a wavelength of 280 nm, hydroxycinnamates at 320 nm, and flavonol glycosides at 360 nm [27]. In another study, HPLC chromatograms recorded at selected wavelengths were used for the detection of: ellagitannins—254 nm, 1-O-cinnamoyl-flavan-3-ols and procyanidins—280 nm, hydroxycinnamic acids—320 nm, ellagic acid and flavonols—360 nm, and anthocyanins—520 nm [28]. In addition, vitamin C and folate can contribute to absorption in the UV region.

In the vis range, the juices obtained directly from fruits exhibited an absorption band with a maximum at about 500 nm and a less intense maximum at about 430 nm. Absorption in the vis region is ascribed to anthocyanin compounds, which are responsible for the characteristic and attractive color of strawberry. A typical UV–vis spectrum of an anthocyanin shows two major absorption bands, the first in the UV region with maxima at a wavelength region between 260 and 280 nm and the other in the vis region with maximums between 490 and 550 nm. An additional band is observed in the wavelength range of 310–340 nm for compounds with acylated sugar moieties [29]. The main anthocyanin present in strawberries is pelargonidin-3-glucoside, which exhibits a band in the vis range with the maximum at about 500 nm and an additional maximum at about 430 nm [30]. The presence of cyanidin-3-glucoside in smaller amounts has been also reported in strawberries. In addition to glucose, rutinose—and, more rarely, arabinose and rhamnose—has been identified as substituting sugars in strawberry anthocyanin [1]. 

The differences in the absorption spectra of the processed beverages compared to the direct juices resemble the different chemical compositions of these two groups of products, which may be due to several factors, including the variety of raw materials, the processing technology, and the storage conditions. The long-wavelength shift in anthocyanin absorption to 525 nm observed for some processed products (Figure 1D) may be an indication of the addition of other fruits or ingredients. In fact, to extend the shelf life of strawberry products and their attractive color, natural pigments (such as chokeberry, elderberry, and black currant) or synthetic pigments are added. On the other hand, the short wavelength shift of the band in the vis range, observed for some products, may be the consequence of the decomposition of strawberry anthocyanins, which are known to be less stable than in many other fruits [27], and which are affected by both processing technology (pasteurization, heat treatment) and storage conditions [31]. In particular, compared to high-intensity pulsed electric field treatment, heat treatments have been shown to negatively impact the shelf life of strawberry juice and increase the browning index and concentration of 5-(hydroxymethyl)-2-furfural [32].

**Figure 2:**
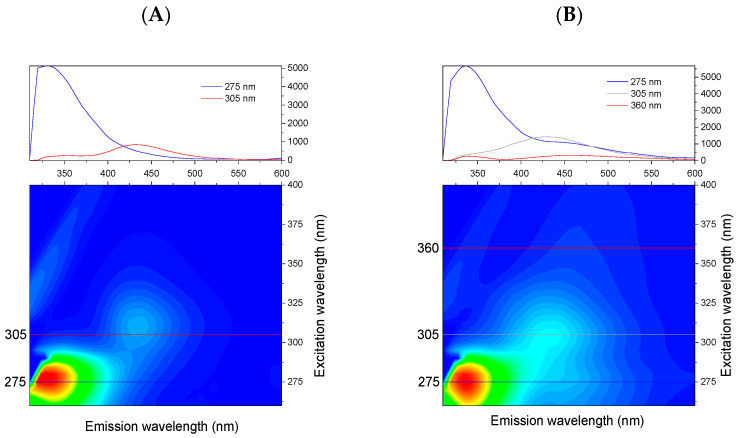
Contour maps of average EEMs for: (**A**) direct juice, (**B**) processed commercial beverages.

Figure 2 shows the contour maps of the average EEMs of two groups of the studied products—direct juices and processed commercial beverages.

For most of the studied products, two distinct emission bands were observed. The first band has an excitation maximum at about 275 nm and an emission maximum in the range of 320–360 nm. For the second band, the excitation maximum is located in the range of 290–320 nm, and the maximum emission occurs in the range of 390–460 nm. The average spectra for the two groups of juices revealed that the fluorescence of processed juices extended to longer excitation and emission wavelengths compared to the direct juices. Based on data from the literature, strawberry beverage emission can be related to various classes of phenolic compounds [3,33,34].

For the detailed characterization of fluorescence properties, we analyzed the EEMs using the PARAFAC method. The aim of the analysis was to extract information about the fluorescent compounds contained in strawberry products. Models with a various number of components (from 3 to 5) were analyzed, and the characteristics of these models are presented in Table S2 (Supplementary Materials). The model with four components was selected as optimal based on the core consistency criteria [24]. A high value of this parameter (93%) indicated the suitability of the selected model. The stability of the four-component model was further validated by means of a split-half analysis, where the data set was divided into half for a comparison of similarity. In the data set studied, the similarity measure of two splits was 76.3%. The parameters of the PARAFAC model with four components are shown in Table 1.

**Figure 3:**
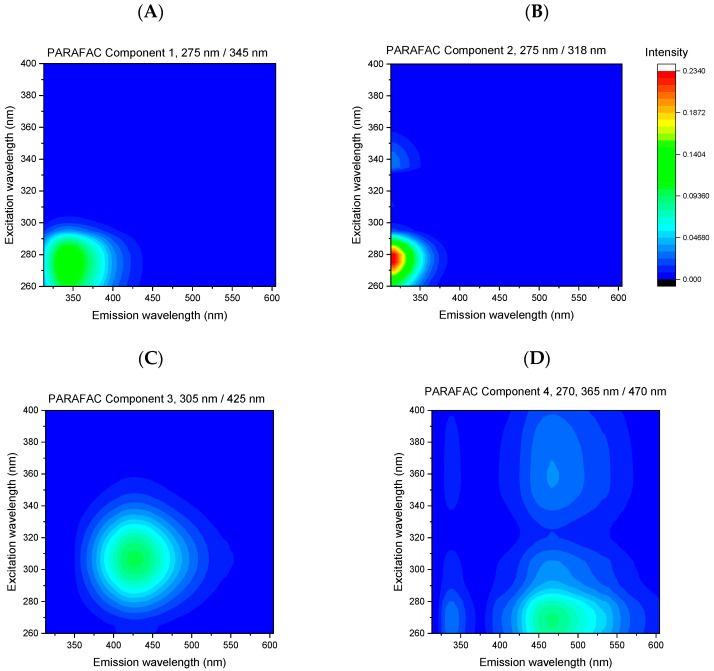
The contour maps of EEMs of fluorescent components obtained as a result of PARAFAC of EEMs of direct fresh juices and processed commercial strawberry beverages: (**A**) component 1, (**B**) component 2, (**C**) component 3, (**D**) component 4.

**Figure 4:**
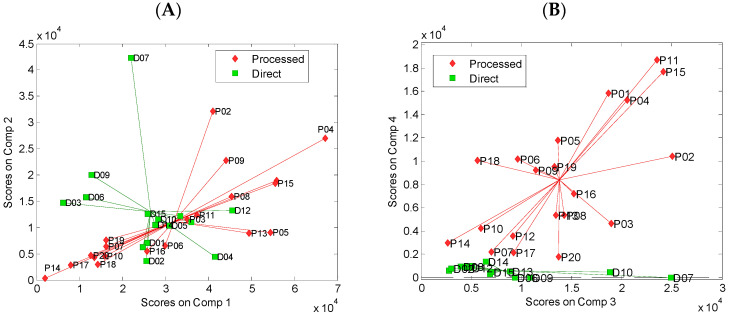
The results of PARAFAC of EEMs of direct fresh and processed commercial strawberry beverages: (**A**) scores for component 1 vs. component 2, (**B**) scores for component 3 vs. component 4.

The four extracted PARAFAC components were characterized by the maxima at the following pairs of excitation and emission wavelengths: component 1—275/345 nm, component 2—275/318 nm, component 3—305/425 nm, and component 4—270, 365/470 nm. The EEMs and relative contributions of these components are presented in Figure 3 and Figure 4.

Due to the diversity of the phenolics present in strawberries, the extracted PARAFAC components correspond more to the group of compounds with a similar structure than to individual components. Only a tentative assignment of the PARAFAC components based on the literature data is proposed. The first component (275/345 nm) can be attributed to hydroxybenzoic acids [35]. The second component (275/318 nm) may correspond to the monomeric flavan-3-ols, in particular, catechin, for which the excitation maximum at 279 nm and the emission maximum at 317 nm were reported [36]. The fluorescence of the third component (305/425 nm) may be related to ellagic acid, which was reported as the dominant phenolic acid in strawberries and has an emission maximum at 425 nm [3,35]. Furthermore, hydroxycinnamic acids, which emit with a maxima in the range of 420–455 nm, may contribute to this component [35]. The fourth component (270, 365/470 nm) may be related to nonenzymatic browning products formed during the thermal processing of food products [37]. It may also be related to quercetin and kaempferol, flavonols that are present in strawberry fruit [17]. It should be emphasized that unambiguous identification of fluorescent components would require the correlation of fluorescent signals with the content of selected components determined with the use of selective methods, e.g., HPLC.

The relative concentrations of the PARAFAC components extracted in individual beverages are shown in Figure 4. For the direct juices and processed commercial strawberry beverages, the score values of the first two fluorescent components varied widely (Figure 4A); however, these compounds do not differentiate the two groups of products. On the other hand, the analysis of component 4 clearly shows that it is present in considerably higher amounts in the processed commercial beverages. On the contrary, all the direct juices have a very low (or zero) concentration of this component (Figure 4B). Based on this observation, it seems that component 4 is related to the compound formed during the thermal processing of juice [37]. The statistical analysis of score values for the two groups of juices revealed significant differences between the mean values of the third and fourth components, Table 1. The mean score values for the first and second components did not differ significantly for both groups of juices.

### 3.2. Principal Component Analysis of Spectral Data

**Figure 5:**
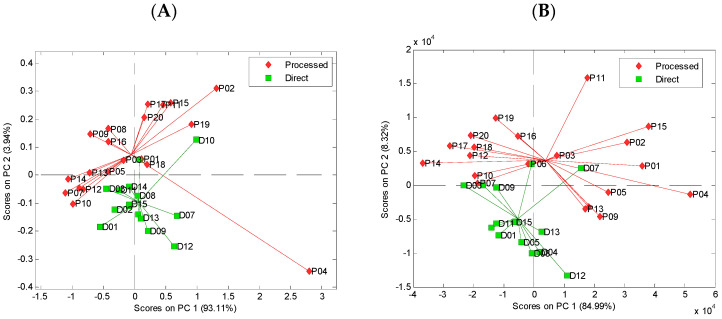
Score plots of PCA analysis of fresh and commercial strawberry juice based on the UV–vis absorption spectra (**A**) and unfolded excitation–emission matrices (EEMs) (**B**).

The exploratory analysis of the absorption spectra and uEEMs using the PCA revealed differences between the direct juices and processed commercial beverages. The results of the PCA performed using UV–vis absorption spectra are presented in Figure 5A. The two groups of beverages are differentiated in the plane defined by PC1 and PC2, which describe 93.11% and 3.94% of the variation of the original spectra, respectively. The results of the PCA carried out on the uEEM are shown in Figure 5B. The grouping of the beverages belonging to two categories of direct juices and processed products is observed in the plane defined by PC1 and PC2. The two first components describe 84.99% and 8.32% of the variation of the original data, respectively.

### 3.3. Classification Models

**Figure 6:**
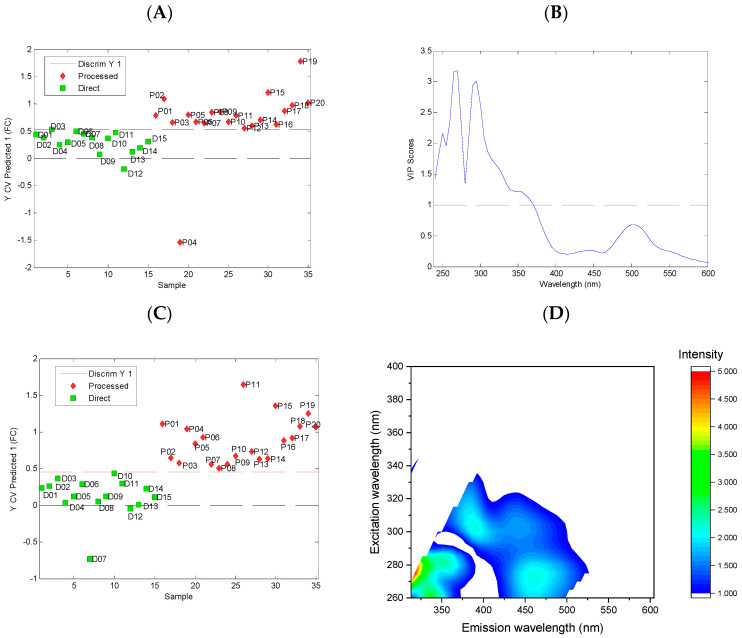
Classification models for direct and processed strawberry juice obtained using PLS-DA method. Models based on UV–vis absorption spectra: classification results (**A**) and variable importance in projection (VIP) plot (**B**). Model based on uEEMs: classification results (**C**) and refolded variable importance in projection (VIP) plot (**D**).

To evaluate the possibility of classifying the tested beverages into two categories (fresh and processed) and to interpret the spectral differences between them, we developed the PLS-DA models. The analyses on the absorption and fluorescence data were performed separately. In the first step, we developed models based on the entire absorption (UV–vis spectra) and fluorescence (entire uEEMs) data. To identify the spectral ranges that significantly contribute to the discrimination between the two studied categories of juices, the variable importance in projection (VIP) method was used. The variables with a significant contribution to the discrimination between the categories studied are characterized by VIP values greater than unity. The classification results and VIP plots for these models are presented in Figure 6.

The PLS-DA model based on UV–vis absorption spectra enabled the correct classification of all the direct juices, while one sample of the processed juice was incorrectly classified, Figure 6A. The analysis of the VIP plot revealed that the spectral range that contributed to the model mainly corresponded to absorption in the UV region.

The PLS-DA model built using entire uEEMs enabled the correct classification of all the studied samples, Figure 6C. The analysis of the VIP plot for this model revealed that the emission in the wide-wavelength range contributed significantly to the discrimination between the fresh and processed juices. The fluorescence below 450 nm may originate mainly from phenolic compounds. The broad emission band between 450 and 500 nm may correspond to the fluorescence of nonenzymatic browning products, which are formed during juice processing and storage [37,38]. 

In the next step, we developed the PLS-DA models using the selected spectral ranges of absorption spectra: UV (240–350 nm) and vis (350–600 nm). Moreover, additional PLS-DA models were built on the basis of fluorescence, including models based on PARAFAC scores and sets of selected emission spectra. The performances of all the classification models are presented in Table 2.

All the models presented exhibited a high classification performance. The best models, which enabled the correct classification of all the samples, were obtained from the analysis of the absorption spectra in the vis region, the analysis of entire uEEMs, and the analysis of the set of emission spectra with excitation at wavelengths of 275, 305, and 365 nm. 

## 4. Discussion

The results of the exploratory analysis of the absorption and fluorescence properties of the studied juices reveal differences, which may be the result of the different composition of raw material and processing applied to commercial beverages. The good classification results obtained using the PLS-DA method prove the significant differences in the absorption and fluorescence properties between the direct and commercial juices. These differences can be the effect of processing, which includes preservation using thermal treatment and the addition of some ingredients. The presence of additional ingredients in commercial products may be the reason for the good classification results. It indicates that spectral analysis can be used for the detection of the presence of some additives. In particular, changes in pigment composition may be easily detected using absorption spectra in the vis region. Thus, absorption spectroscopy could potentially be used for the detection of the addition of synthetic pigments or other fruits containing natural pigments. On the other hand, this method could be used for monitoring natural strawberry-pigment decomposition during processing and storage. 

Fluorescence seems to be suitable for controlling the thermal treatment. Despite the diversity of the composition of the commercial beverages studied, all the samples in this category were differentiated from the direct juice by a higher content of fluorescent component 4 extracted by the PARAFAC analysis. Because a common feature of all the commercial juices was preservation by pasteurization, this component appears to be formed during thermal treatment. In fact, the formation of fluorescent products of nonenzymatic browning was observed during the thermal treatment of apple juice [37]. Thus, the results presented indicate that fluorescence may also be useful for monitoring the thermal processing of strawberry juice.

## 5. Conclusions

The A-TEEM^TM^ technique provides the general characteristics of the natural chromophores and fluorophores present in strawberry beverages. The analysis of the EEMs using the PARAFAC enabled the characterization of four fluorescent components and revealed some differences among the fluorescence of direct juices and commercial beverages. The differences in the absorption and fluorescence properties of these two groups of juices were further confirmed by the PCA analysis. These two classes of products were successfully classified on the basis of their absorption and fluorescence using the PLS-DA method. 

These results may be potentially useful for the development of rapid methods for the processing and authenticity control of strawberry beverages. However, further studies are needed with a larger number of samples and defined processing technology to develop reliable models for particular applications and to validate these models with the external samples sets. The presented results demonstrate that the A-TEEM^TM^ technique may be a useful tool for processing and authenticity control in the beverage industry, enabling the rapid analysis of a large number of samples.

## Figures and Tables

**Table 1:** Results of parallel factor analysis (PARAFAC) of EEMs of strawberry beverages.

Component No.	λ_exc_/λ_em_	Mean of Scores Value for Two Classes
Component 1	275/345 nm	Direct: 26,552 ± 10,675; Processed: 31,983 ± 19,104 *
Component 2	275/318 nm	Direct: 13,051 ± 9164; Processed: 11,129 ± 8727 *
Component 3	305/425 nm	Direct: 8125 ± 6162; Processed:13,771 ± 6447 **
Component 4	270, 365/470 nm	Direct: 615 ± 418; Processed: 8403 ± 5346 **

* Means are not significantly different at 0.05 level; ** Means are significantly different at 0.05 level.

**Table 2:** Performance of classification models for direct and processed strawberry beverages developed using absorption and fluorescence spectra.

Model	LV	Misclassification Error	Sensitivity for Class 1	Specificity for Class 1
Absorption spectra (UV–vis)	3	0.029	0.93	1.00
Absorption spectra UV (240–350 nm)	3	0.057	0.93	0.95
Absorption spectra vis (350–600 nm)	5	0	1.00	1.00
PARAFAC-EEMs	2	0.057	0.93	0.95
uEEMs	4	0	1.00	1.00
Emission spectra with excitation at 275, 305, 365 nm	4	0	1.00	1.00
Emission spectra with excitation EM 305, 365 nm	6	0.086	1	0.85

## Data Availability

Not applicable.

## Supplementary Materials

The following supporting information can be downloaded at: https://www.mdpi.com/article/10.3390/foods11142143/s1, Table S1: Characteristics of the studied samples; Table S2: Characteristics of PARAFAC models of EEMs of strawberry beverages.
