# Supplementary material for: Characterization and Classification of Direct and Commercial Strawberry Beverages Using Absorbance–Transmission and Fluorescence Excitation–Emission Matrix Technique
Source: Foods. 2022 Jul 20;11(14):2143. doi: 10.3390/foods11142143 (PMC9323525; doi:10.3390/foods11142143)
Supplement: Supplementary file 1 [file foods-11-02143-s001.zip › foods-1761803-supplementary.pdf]

Table S1. Characteristics of the studied samples.

**D1**	Azja, 2018
**D2**	Azja, 2018
**D3**	Azja, 2019
**D4**	Azja, 2019
**D5**	Azja, 2019
**D6**	Florence, 2018
**D7**	Florence, 2018
**D8**	Florence, 2019
**D9**	Florentina, 2019
**D10**	Honeoye, 2018
**D11**	Korona, 2018
**D12**	Romina, 2018
**D13**	Rumba, 2018
**D14**	Rumba, 2019
**D15**	Rumba, 2019
**P1**	Juice:100% strawberry puree juice
**P2**	Juice: 100% strawberry juice
**P3**	Juice: 50% juice made of strawberries, 50% sugar
**P4**	Juice: Organic strawberry juice, 50% juice made of strawberries, 50% sugar
**P5**	Nectar: 65% juice, made of strawberries, water, sugar
**P6**	Drink: 40% strawberry puree juice, water, sugar, an acidity regulator, aroma, antioxidant
**P7**	Juice: 20% strawberry juice, 80% apple juice
**P8**	Juice: 50% strawberry juice, 50% apple juice
**P9**	Drink/juice: Strawberry 31%, Bananas 24%, Apples 23%, Oranges 16%, Aronia 3%, Raspberries 3%
**P10**	Drink/juice: 19% strawberry puree, 9% apple, 1% chokeberry, 1% black currant, aromas
**P11**	Syrup: 51% juice, made of strawberries, sugar
**P12**	Syrup: 100% strawberry juice
**P13**	Syrup: 100% strawberry low-sugar syrup, 64% strawberry juice, 34% sugar
**P14**	Syrup: 53% juice, made of strawberries, sugar, citric acid
**P15**	Syrup: 53% juice made of strawberries, sugar, citric acid
**P16**	Syrup: 50% juice, made of strawberries, 50% sugar
**P17**	Syrup: 1.85% of concentrated strawberry juice, an acidity regulator, water, aroma, antioxidant, sugar
**P18**	Syrup: 1.8% of concentrated strawberry juice, an acidity regulator, water, aroma, antioxidant, sugar, other concentrated juices
**P19**	Syrup: 0.25% of concentrated strawberry juice, an acidity regulator, water, other juices
**P20**	Syrup: 0.2% of concentrated strawberry juice, an acidity regulator, water, aroma, antioxidant, sugar, other concentrated juices

Table S2.

Characteristics of PARAFAC models of EEMs of strawberry beverages

Number of components	Spectral profiles of components $\lambda_{ex}/\lambda_{em}$ (nm)	Explained variance (%)	Core consistency	Split half analysis - similarity measure (%)
3	275, 365/345<br>277,322/310<br>310,385/435	98.627	92	1.3
4	275/345 nm<br>275/318 nm<br>305 nm/425 nm<br>270, 365 nm/470 nm	99.179	93	76.3
5	275/345<br>277/310<br>316/333<br>305/425<br>270, 365/465	99.348	91	0.4
